# Disseminated blastomycosis with cutaneous involvement in a young man in Ottawa, Canada

**DOI:** 10.1093/skinhd/vzae016

**Published:** 2025-01-22

**Authors:** Jaime N Turk, Laura D Chin, Stephanie L Petkiewicz, Steven J Glassman

**Affiliations:** Department of Dermatology, University of Ottawa, Ottawa, ON, Canada; Department of Dermatology, University of Ottawa, Ottawa, ON, Canada; Department of Dermatology, University of Ottawa, Ottawa, ON, Canada; Department of Dermatology, University of Ottawa, Ottawa, ON, Canada

Dear Editor, Blastomycosis is a deep fungal infection caused by *Blastomyces dermatitidis*, typically starting in the lungs and potentially spreading to the skin.^1^ Diagnosis of skin lesions is challenging due to similarity to inflammatory and infectious dermatoses.^2^ A healthy 26-year-old man with a cough and pleuritic pain showed consolidation on X-ray and was initially treated for pneumonia. One month later, he developed crusted verrucous plaques (Figure 1a). Biopsy revealed fungal organisms in giant cells (Figure 1b), highlighted by silver staining (Figure 1c). He was treated with itraconazole for 6 months with follow-up. Early recognition and treatment are essential in preventing complications of blastomycosis.

**Figure 1:**
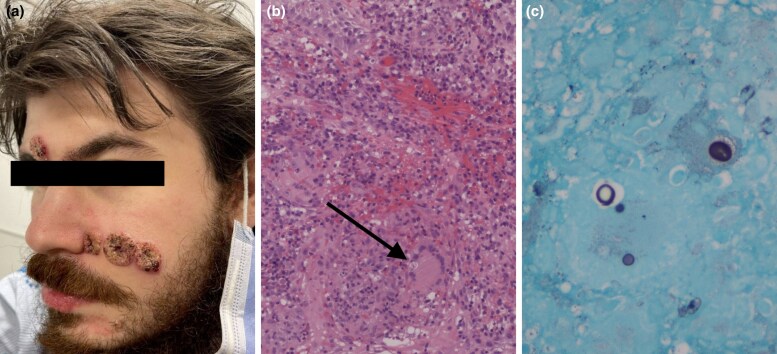
Clinical and histopathological findings in a patient with cutaneous blastomycosis. (a) Verrucous crusted plaques on the face show a cutaneous presentation of blastomycosis. (b) Haematoxylin and eosin staining shows pseudoepitheliomatous hyperplasia with mixed inflammatory infiltrates including multinucleated giant cells. The arrow points toward a fungal organism within the giant cell. (c) Silver staining highlights fungal organisms within histiocytes.

## Data Availability

No new data were generated or analysed in support of this research.
